# Highly Pathogenic Avian Influenza H5 Hemagglutinin Fused with the A Subunit of Type IIb *Escherichia coli* Heat Labile Enterotoxin Elicited Protective Immunity and Neutralization by Intranasal Immunization in Mouse and Chicken Models

**DOI:** 10.3390/vaccines7040193

**Published:** 2019-11-22

**Authors:** Neos Tang, Shi-Wei Lin, Ting-Hsuan Chen, Jia-Tsrong Jan, Hung-Yi Wu, Suh-Chin Wu

**Affiliations:** 1 Institute of Biotechnology, National Tsing Hua University, Hsinchu 30013, Taiwan; tomtang1107@gmail.com (N.T.); wxes9350409@gmail.com (S.-W.L.); sadam1114@gmail.com (T.-H.C.); 2 Genomics Research Center, Academia Sinica, Taipei 11529, Taiwan; tsrong33@gate.sinica.edu.tw; 3 Graduate Institute of Veterinary Pathobiology, National Chung Hsing University, Taichung 402, Taiwan; hwu2@dragon.nchu.edu.tw; 4 Department of Medical Science, National Tsing Hua University, Hsinchu 30013, Taiwan

**Keywords:** type IIb heat labile enterotoxin, mucosal vaccine, H5N1 vaccine

## Abstract

Highly pathogenic avian influenza viruses are classified by the World Organization for Animal Health (OIE) as causes of devastating avian diseases. This study aimed to develop type IIb *Escherichia* *coli* heat-labile enterotoxin (LTIIb) as novel mucosal adjuvants for mucosal vaccine development. The fusion protein of H5 and LTIIb-A subunit was expressed and purified for mouse and chicken intranasal immunizations. Intranasal immunization with the H5-LTIIb-A fusion protein in mice elicited potent neutralizing antibodies in sera and bronchoalveolar lavage fluids, induced stronger Th1 and Th17 cellular responses in spleen and cervical lymph nodes, and improved protection against H5N1 influenza virus challenge. More interestingly, intranasal immunization with the H5-LTIIb-A fusion protein in chickens elicited high titers of IgY, IgA, hemagglutinin inhibition (HAI), and neutralizing antibodies in their antisera. This study employed the novel adjuvants of LTIIb for the development of a new generation of mucosal vaccines against highly pathogenic avian influenza viruses.

## 1. Introduction

Influenza A virus is a member of the Orthomyxoviridae family and causes several pandemic infections, such as 1918 H1N1 (Spanish flu), 1957 H2N2 (Asian flu), 1968 H3N2 (Hong Kong flu), 1977 H1N1 (Russian flu), and 2009 H1N1 pandemics [1]. Highly pathogenic avian influenza (HPAI) H5N1 viruses can cause serious infection in humans. Infection with HPAI H5N1 results from contact with infected avians; however, these viruses cannot be transmitted efficiently among humans. HPAI H5N1 viruses mainly infect the lower respiratory tracts and result in lung damage and plasma cytokine dysregulation [2,3]. In 1997, the first case of human infection with HPAI H5N1 virus occurred in Hong Kong; the virus re-emerged in 2003, and since then, human infections caused by it have been continuously reported in Asia, the Middle East, Europe, and Africa, with an approximately 60% mortality rate [1,4,5,6].

The route of delivery determines the area protected by a mucosal vaccine [4]. Mucosal immune responses can be elicited by several routes, including nasal, sublingual, oral, rectal, vaginal, and trans-dermal routes. For airway protection, nasal vaccine delivery provides a major defense and generates antigen-specific mucosal immunity as well as systemic immunity against foreign antigens [7,8]. Antigens can be recognized in nasopharynx-associated lymphoid tissues (NALTs) that contain M cells, antigen presenting cells, T cells, and B cells to trigger mucosal immune responses, such as IgA-secreting B plasma cells. Nasal delivery of vaccines generally requires the use of adjuvants to generate effective mucosal immunity [8]. The most potent mucosal adjuvants are derived from three bacterial products: (i) ADP-ribosylating enterotoxins, such as cholera toxin (CT) and the heat-labile enterotoxin from *Escherichia coli* (LT); (ii) unmethylated CpG dinucleotides; and (iii) monophosphoryl lipid A (MPL) [9]. Other TLR agonists, such as poly(I:C), Pam3CSK4, and R848 (Resiquimod), have also been reported to induce potent mucosal adjuvanticity [10]. The ADP-ribosylating enterotoxins CT and LT (or their detoxified mutants) and the TLR-agonists represent two different classes of adjuvants that may be used individually or in combination (for the potential synergy) for mucosal adjuvant development [9]. The ADP-ribosylating enterotoxins, such as CT and LT, are the most typical adjuvants used for mucosal vaccine development [9,10,11].

LT is a member of the AB-class of bacterial toxins; it is composed of an enzymatically active A subunit with ADP-ribosyltransferase activity and a B subunit that mediates binding to eukaryotic cell surfaces [9]. The A subunit of LT catalyzes the activation of adenylate cyclase by raising cAMP levels via ADP-ribosylation. The B subunit of LT, produced as a pentamer, binds to the cellular receptors of glycolipids (mainly for gangliosides) or other glycoproteins for transporting the A toxin-subunit into the cells. The LT family can be divided into type I and type II subfamilies on the basis of the amino acid differences in their B subunits, accounting for differential ganglioside-receptor binding profiles [12]. The B subunits of LT-IIa and LT-IIb bind to TLR2 and activate TLR2-mediated cellular activation [13,14]. LTI-B and LT-II (AB5-holotoxin) are more potent in their adjuvanticity for mucosal immunity [7,9]. The LTIIb-B5 subunit activates immune responses through the TLR-2/1-dependent activation of pattern recognition receptors [12,15]. 

Both LTI and LTII are composed of an A polypeptide that is non-covalently linked to a pentameric ring of the B subunit [9]. The A subunit after proteolytic cleavage and disulfide bond reduction separates into the enzymatically active A1 subunit and a smaller A2 peptide. Transport of A1 into the cytoplasm triggers ADP-ribosylation of the intracellular GSα regulatory protein to induce constitutive activation of the cell’s adenylate cyclase and causes dysregulation of cAMP-sensitive ion transport mechanisms, inhibiting intracellular salt absorption, increasing electrolyte transport into the gut lumen [16], and creating an osmotic gradient favoring intestinal water secretion [17]. Direct fusion of toxoid antigen to the A subunit at the N- or C-terminus, or inside the A subunit of LTR192G, has been reported to neutralize antitoxin antibodies, which suggested the application of toxoid fusions in ETEC vaccine development [18]. Moreover, the A1 domain of cholera toxin (CTA1) fused with influenza A virus matrix protein 2 (M2e) and immunoglobulin binding D domain (DD) to CTA1-M2e-DD conferred a broad range of protective immunity against homologous and heterologous influenza A viruses [19,20]. We, thus, investigated the use of the LTIIb-A subunit directly fused to the H5 antigen as a single associated antigen-adjuvant molecule for developing mucosal H5N1 avian influenza vaccines for poultry use. Because the AB5 structure of LTIIb is composed of the A subunit (LTIIb-A) and the pentameic B subunits (LTIIb-B5) [9], the B subunit was considered as a non-toxic and more safer mucosal adjuvant [21]. As the LT AB5-holotoxins are believed to provide more potent adjuvanticity [7,9], we also investigated the use of H5-LTIIb-A mixed with the LTIIb-B5 protein for intranasal vaccinations in mice and chickens. Our study employed the novel adjuvants of LTIIb for the development of a new generation of mucosal vaccines against HPAI H5N1 viruses.

## 2. Materials and Methods

### 2.1. H5-LTIIb-A Fusion Protein and Recombinant H5 Protein Cloning, Expression, and Purification 

The H5 ectodomain gene was derived from the influenza A virus H5N1 subtype (A/Thailand/1(KAN-1)/2004 strain), and the LTIIb-A gene was derived from the sequence of A chain of heat labile enterotoxin IIb (accession no. P43528.2). These two genes were co-cloned with an N-terminal sixfold histidine tag, a GC N4 trimerization domain, and a glycine–serine linker (GGSGGGSG) into a pFastBac plasmid.

Recombinant H5 proteins were generated as previously described [22,23]. For H5-LTIIb-A fusion protein and recombinant H5 protein production, Sf9 cells were grown in SF900-II medium (Invitrogen) and infected with recombinant baculoviruses at 2 × 10^6^ cells/mL for 48 h. Proteins were purified by nickel affinity chromatography and eluted with 50%–100% Buffer B. Purified proteins were concentrated using 30 kDa-restricted centrifugal filters (Millipore), dialyzed with phosphate buffered saline (PBS), and stored at −20 °C.

### 2.2. Recombinant LTIIb-B5 Cloning, Expression, and Purification 

The codon-optimized type IIb heat labile enterotoxin B subunit (LTIIb-B5) gene (accession no. P43529) was cloned into a pET22b (+) expression vector with a C-terminal sixfold His-tag. LTIIb-B5-pET22b (+) plasmids were transformed into *E. coli* BL21 cells (DE3) (Invitrogen), which were cultured in Luria-Bertani (LB) broth. Recombinant proteins were produced 4 h after 1 mM IPTG stimulation. Cells were collected by centrifugation at 5000 rpm for 15 min at 4 °C. Pellets were resuspended in 40 mL buffer A (300 mM Tris, 50 mM NaCl, 10 mM imidazole, 5% glycerol; pH 7.2) with 1 mM PMSF (USB) for purification. Cells were homogenized at 15k PSI. Inclusion bodies were solubilized with 8 M urea, eluted with 30%–40% Buffer B (300 mM Tris, 50 mM NaCl, 500 mM imidazole, 5% glycerol; pH 7.2) by nickel affinity chromatography column, and dialyzed with 1× PBS overnight. Purified proteins were concentrated using 3 kDa centrifugal filters (MILLPORE) and passed through an endotoxin removal column (Cellfine). Limulus amoebocyte assay kit was used to measure the residual LPS content of LTIIb-B5.

### 2.3. Functional TLR Ligand Assays

HEK 293A cells were seeded and incubated overnight in culture dishes (6 × 10^6^ cells/dish) prior to co-transfection with pDUO-hTLR1/hTLR2 plasmids (InvivoGen) (7.5 μg/dish) and pGL4.32 (luc2p/NF-κB-RE/Hygro) vectors (Promega) (3 μg/dish) using the Turbofect (Fermentas) transfection reagent. Transfected cells were seeded into 96-well plates at a density of 5 × 10^4^ cells/well. The following day, H5, H5-LTIIb-A, LTIIb-B5, or Pam3CSK4 (InVivoGen) were serially diluted (10 μg/mL to 1 pg/mL) and were used to treat batches of cells for 5 h. Treated cells were lysed with cell lysis buffer (Glo-lysis buffer; Promega). Luciferase activity was determined by adding a luminescent substrate (neolite assay; Perkin Elmer) and measuring the relative luminescence unit (RLU) of each well at a wavelength of 560 nm using a Victor II microplate reader (both PerkinElmer).

### 2.4. Hemagglutination Assay

Twofold serial dilution of protein samples was performed with PBS (pH 7.4) at a starting concentration of 70 μg/mL. Then, the serially diluted protein samples were added to a 96-well V-plate at 50 μL/well, and PBS containing 0.5% turkey erythrocytes was then added at 50 μL/well. The plate was incubated at room temperature for 30 min for hemagglutination. The titer of the recombinant protein was defined as the maximum dilution at which erythrocyte precipitation was observed for the first time.

### 2.5. Fetuin Binding Assay

The binding solution (0.05 M carbonate buffer solution, pH 9.6) containing 100 μg/mL fetuin was added to a 96-well plate at 100 μL/well. After incubating at 4 °C for 16–18 h, the plate was washed with PBS containing 0.05% Tween-20 (referred to as PBST). PBS (200 μL) containing 1% bovine serum albumin (BSA) was then added for blocking at room temperature for 1 h to prevent non-specific binding. Next, the 96-well plate was washed with PBST, and serially diluted protein samples (twofold serial dilution prepared with PBS containing 1% BSA and 0.05% Tween-20 at a starting concentration of 10 μg/mL) were added to each well. The plate was incubated at room temperature for 1 h and washed with PBST. Then, the 96-well plate was treated with anti-H5 hemagglutinin antibody at 100 μL/well (1:10,000 dilution) for 1 h, washed with PBST, and treated with HRP-conjugated anti-rabbit IgG secondary antibody at 100 μL/well (1:5000 dilution) for 1 h at room temperature. Finally, HRP chemiluminescence substrate, 3,3’,5,5’-tetramethylbenzidine (TMB; Biolegend), was added to the 96-well plate at 100 μL/well. After color development in the dark for 15 min, 2 N sulfuric acid was added at 100 μL/well to stop the reaction, and the absorbance of each well was measured at 450 nm (O.D. 450) using an ELISA reader (TECAN SUNRISETM).

### 2.6. Mouse and Chicken Immunization and Sample Collection 

Groups of female BALB/c mice (6–8 weeks old) purchased from the National Laboratory Animal Center, Taiwan, were immunized with 2.5, 5, or 10 μg H5 or H5-LTIIb-A with or without 5 μg LTIIb-B5 proteins. For intranasal immunizations, mice were anesthetized using isoflurane (Panion and BF Biotech) by inhalation prior to the application of 20–30 μL immunization samples or a control. All mouse groups were immunized three times (at week 0, 3, and 6); serum samples were collected at week 8. Most mice were sacrificed at week 9 and splenocytes, cervical lymph notes (CLNs), and bronchoalveolar lavage fluids (BALFs) were collected. Groups of chickens (three per group) were administered, via intranasal injection, three doses of the H5-LTIIb-A fusion protein (10 μg), the recombinant H5 protein (10 μg), the H5-LTIIb-A fusion protein (10 μg) in combination with the recombinant LTIIb-B5 protein (5 μg), or PBS (as sham control). The time interval between each of the immunizations was approximately two weeks. Serum samples were collected two weeks after the third dose immunizations. All samples were stored at −20 °C until used for further analyses. All procedures involving mice were performed in accordance with the guidelines established by the Laboratory Animal Center of National Tsing Hua University (NTHU). All procedures involving chickens were performed in accordance with the guidelines established by the Laboratory Animal Center of National Chung Hsin University (NCHU). Animal protocols were reviewed and approved by the NTHU (IACUC no.: 10636) and the NCHU (IACUC no.: 106-009) Institutional Animal Care and Use Committees.

### 2.7. Hemagglutination (HA)-Specific Antibody Titer Analysis

ELISAs were used to measure antibodies in immunized mouse sera and BALF samples. After coating with 2 μg/mL recombinant HA protein, 96-well ELISA plates (Costar) were incubated overnight at 4 °C, blocked with 1% BSA in PBS at room temperature, and incubated for 30 min. Serially diluted samples were added to each plate and incubated for 1 h at room temperature. Next, HRP-conjugated goat anti-mouse IgG antibodies (1:30,000) or HRP-conjugated goat anti-mouse IgA antibodies (1:10,000) (Bethyl) were added to individual plates and incubated for another 1 h at room temperature. TMB substrates (BioLegend) were added for coloration, followed by incubation for 15 to 20 min at room temperature; reactions were stopped with 2 N H_2_SO_4_ and detected using an ELISA reader (OD_450_). End-point titers were measured as fourfold absorbance of a negative control.

### 2.8. Neutralization Assay Using H5N1 Pseudotyped Particles (H5N1pp)

Neutralizing antibodies were quantified as reduced luciferase expression levels following H5N1pp transduction in MDCK cells [24,25]. Briefly, HEK293T cells were co-transfected with pNL-Luc-E^-^R^-^ plasmid (HIV backbone) and pcDNA3.1(+) expressing HA from the A/Thailand/1(KAN-1)/2004 strain and pcDNA4B expressing the NA of the A/VietNam/1203/2004 strain. Culture supernatants were collected and concentrated 48 h post-transfection. H5N1pp titer was determined by p24 ELISA (Clontech). Next, 50 µL H5N1pp (50 TCID_50_) was incubated with 50 µL diluted antisera (two-fold serial dilution starting from 1:40) for 1 h at 37 °C, followed by the addition of MDCK cells (1.5 × 10^4^ cells/well). Cells were lysed with cell lysis buffer (Glo-lysis buffer; Promega) after 2 days post-infection. Luciferase activity was measured following the addition of luminescent substrate (neolite assay; Perkin Elmer). Neutralization titers (IC50) were measured as the reciprocal of the serum dilution required to obtain a 50% reduction in RLU compared to a control containing the H5N1pp virus only. Neutralization curves and IC_50_ values were analyzed using GraphPad Prism 5 software.

### 2.9. Cytokine Analysis

Spleen (SPL) and cervical lymph node (CLN) cells were seeded into 96-well plates (5 × 10^5^ cells/well) and stimulated with 1 μg/mL pooled HA peptides (15 mer overlapped by eight amino acids spanning the HA_1_ of A/Viet Nam/1203/2004/H5N1) under 5% CO_2_ for 3 days at 37 °C. Cultured supernatants were collected and stored at −20 °C. ELISA plates (96-well) were coated with IFN-γ, IL-4, or IL-17A capture antibodies, incubated overnight at 4 °C, and blocked with 1% BSA, according to the manufacturer’s instructions (BioLegend). Diluted samples were incubated with capture antibodies for an additional 2 h at room temperature. Cytokines were detected using specific antibodies for 1 h each and interacted with avidin-HRP for 30 min prior to determination of coloration and end-point titers.

### 2.10. Virus Challenges

Groups of BALB/c mice were immunized with three doses of recombinant H5 protein or H5-LTIIb-A fusion protein without or with the LTIIb-B5 adjuvant or three doses of PBS as a sham control. Immunized mice were challenged intranasally with H5N1 NIBRG-14 virus (A/Vietnam/1194/2004) (H5N1) at a 10-fold mouse median lethal dose. Survival rates and body weights were recorded daily for 14 days.

### 2.11. Viral Hemagglutinin Inhibition (HI) Assay

Prior to the assay, 10 μL chicken serum was treated with 30 μL receptor destroying enzyme (RDE; Denka Seiken) at 37 °C for 18–20 h to remove the materials causing nonspecific erythrocyte aggregation. Thereafter, the serum–enzyme mixture was heated at 56 °C for 30 min to eliminate the RDE activity, followed by the addition of 60 μL PBS to the mixture, resulting in a final volume of 100 μL. The 100 μL serum mixture was incubated at 4 °C for 1 h with 5 μL PBS containing 0.5% turkey erythrocytes, and serum supernatant was collected from the mixture by centrifugation (400 × *g*, 10 min, 4 °C). After the serum supernatant was twofold serially diluted with PBS, 25 μL of each diluted solution was mixed at an equal volume with four hemagglutination units (HA unit) of H5N1 NIBRG-14 virus, Baz et al 2013, and incubated at 37 °C for 30 min. Then, 50 μL of 0.5% turkey erythrocytes was added, and hemagglutination was examined after the mixture was incubated at room temperature for 30–60 min.

### 2.12. laque Reduction Neutralization Test (PRNT)

Chicken serum was twofold serially diluted in minimum essential medium-α (MEM-α; Gibco), and 20 μL of each diluted solution was mixed at equal volume with 100 plaque forming units (PFUs) of H5N1 NIBRG-14 virus and incubated at 37 °C for 1 h. The serum–virus mixture and 960 μL of the MEM-α medium containing 0.5 μg/mL TPCK (*N*-tosyl-L-phenylalanine chloromethyl ketone)-treated trypsin (Sigma) were added to 6-well plates seeded with 9.5 × 10^5^ MDCK cells/well and incubated at 37 °C for 1 h. The cells in each well were washed with PBS and covered by 3 mL of MEM-α medium containing 0.5 μg/mL TPCK-treated trypsin and 0.5% agar. After incubation at 37 °C for 48 h, the cells were fixed with 4% paraformaldehyde (Sigma) for 8 h, stained with 1% crystal violet (Sigma) in 20% formaldehyde for 8 h, and destained with water for viral plaque counting. The number of viral plaques reduced after treatment with the serum–virus mixture, compared to the number of viral plaques in the control group treated only with virus, was used to calculate the neutralization percentage and obtain the neutralization curves.

### 2.13. Statistical Analyses

All data in this study were calculated using GraphPad Prism V6.01. Two-tailed Student’s *t*-tests and one-way ANOVA analysis with Tukey’s multiple comparison tests were used to analyze all results except the survival data. Kaplan–Meier analysis was adopted for survival analysis. Statistical significance in all results was expressed as the following: * *p* < 0.05; ** *p* < 0.01; *** *p* < 0.001; **** *p* < 0.0001.

## 3. Results

### 3.1. Expression and Purification of H5-LTIIb-A Fusion Protein

**Figure 1:**
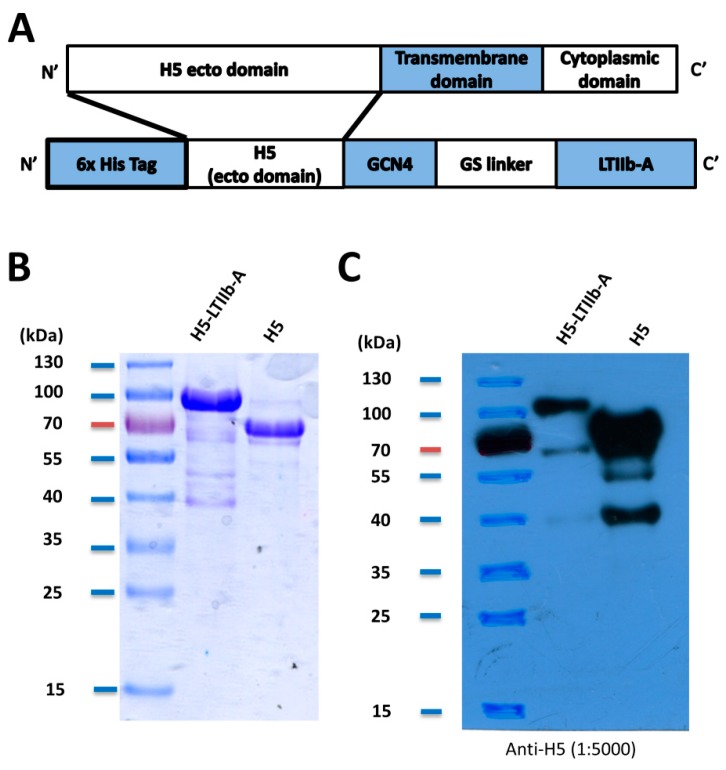
(**A**) A construction scheme illustrating the H5-LTIIb-A fusion protein, which includes a N-terminal His-tag, an ectodomain of H5 hemagglutinin, a GCN4 trimerization motif, a GS linker, and a Type IIb *Escherichia coli* heat-labile enterotoxin A subunit (referred to as LTIIb-A); (**B**) SDS-PAGE gels for the purified H5-LTIIb-A fusion protein and a recombinant H5 protein; (**C**) detection of the H5-LTIIb-A fusion protein and the recombinant H5 protein by western blotting using anti-H5 hemagglutinin antibody.

The sequence of the H5-LTIIb-A fusion protein, which consisted of an H5 ectodomain, a full-length wild-type LTIIb-A, a GCN4 trimerization domain, a GS linker, and a 6-repeated histidine tag, was inserted into a pFastBac plasmid for development of a baculoviral expression system (Figure 1A). After purification by nickel-affinity chromatography, purified H5-LTIIb-A fusion protein was characterized by SDS-PAGE using Coomassie blue staining and western blotting with anti-H5 antibody. The H5 ectodomain (~70 kDa) fused with LTIIb-A (~28 kDa) and other additional domains were observed as the major band at about 100 kDa in the SDS-PAGE gel (Figure 1B) and the western blot (Figure 1C). The fusion protein also exhibited similar H5 epitopes and structural features as observed using polyclonal anti-H5 antibodies. 

### 3.2. Functional and Immunogenic Analysis of H5-LTIIb-A Fusion Proteins

**Figure 2:**
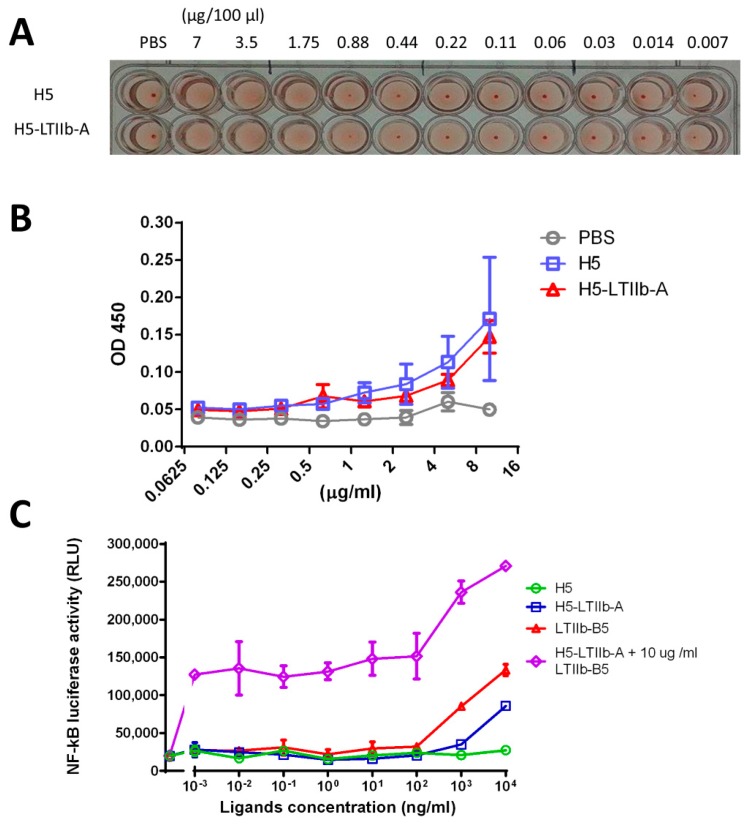
(**A**) Comparison of hemagglutination due to the recombinant H5 protein or the H5-LTIIb-A fusion protein by using hemagglutination assay; (**B**) fetuin binding capability of the recombinant H5 protein and the H5-LTIIb-A fusion protein by using fetuin binding assay; (**C**) detection of the TLR2/1 and NF-κB activation by various protein samples using TLR2/1 functional assay; the various protein samples include the recombinant H5 protein, the H5-LTIIb-A fusion protein, a recombinant protein of *E. coli* type IIb heat-labile enterotoxin B subunit (LTIIb-B5), and the combination of the H5-LTIIb-A fusion protein and 10^4^ ng/ml of the recombinant LTIIb-B5 protein (denoted as H5-LTIIb-A + 10 μg/ml LTIIb-B5).

To determine whether H5-LTIIb-A proteins retain the abilities of hemagglutination and sialic acid-binding as recombinant H5 proteins, the fusion proteins were employed for the hemagglutination assay and fetuin binding assay. In the hemagglutination assay, either the H5-LTIIb-A fusion proteins or the recombinant H5 proteins agglutinated erythrocytes at concentrations above 1.75 μg/100 μL, indicating that the H5-LTIIb-A fusion proteins and the recombinant H5 proteins had a comparable ability to bind sialic acid on erythrocytes and, thus, showed the same binding ability for agglutinating erythrocytes (Figure 2A). In fetuin binding assay, increase of H5-LTIIb-A rose the absorbance to a similar degree as recombinant H5 protein by binding with fetuin, indicating that the two proteins had similar fetuin binding capability (Figure 2B). These results demonstrated that the fusion of H5 and LTIIb-A did not alter the properties of H5.

The TLR2/1 functional assay was performed to assess the effect of the H5-LTIIb-A fusion protein on the TLR2/1 signaling pathway, particularly with LTIIb-B5 (Figure 2C). As we already reported, LTIIb-B5 significantly induced NF-κB signaling via the TLR2/1 receptor in a dose-dependent manner. Treatment with 10^4^ ng/mL recombinant H5 protein did not induce the level of NF-κB signaling as in untreated cells. Intriguingly, 10^4^ ng/mL of H5-LTIIb-A fusion protein alone also triggered the NF-κB signaling, which exhibited a fourfold increase in luminescence intensity compared to the background value. Furthermore, co-treatment with 10^4^ ng/mL H5-LTIIb-A fusion protein and 10^4^ ng/mL recombinant LTIIb-B5 protein elicited NF-κB signaling more prominently compared to that of cells treated with 10^4^ ng/mL H5-LTIIb-A fusion protein alone or 10^4^ ng/mL recombinant LTIIb-B5 protein. These results revealed that the H5-LTIIb-A fusion protein was able to stimulate TLR2/1 receptor to promote the production of NF-κB-driven luciferase. Moreover, the combined use of the H5-LTIIb-A fusion protein and the recombinant LTIIb-B5 protein induced the most significant TLR2/1 activating effect.

### 3.3. Intranasal Immunization with H5-LTIIb-A Elicited High-Titer Antibody Response in Mice

**Figure 3:**
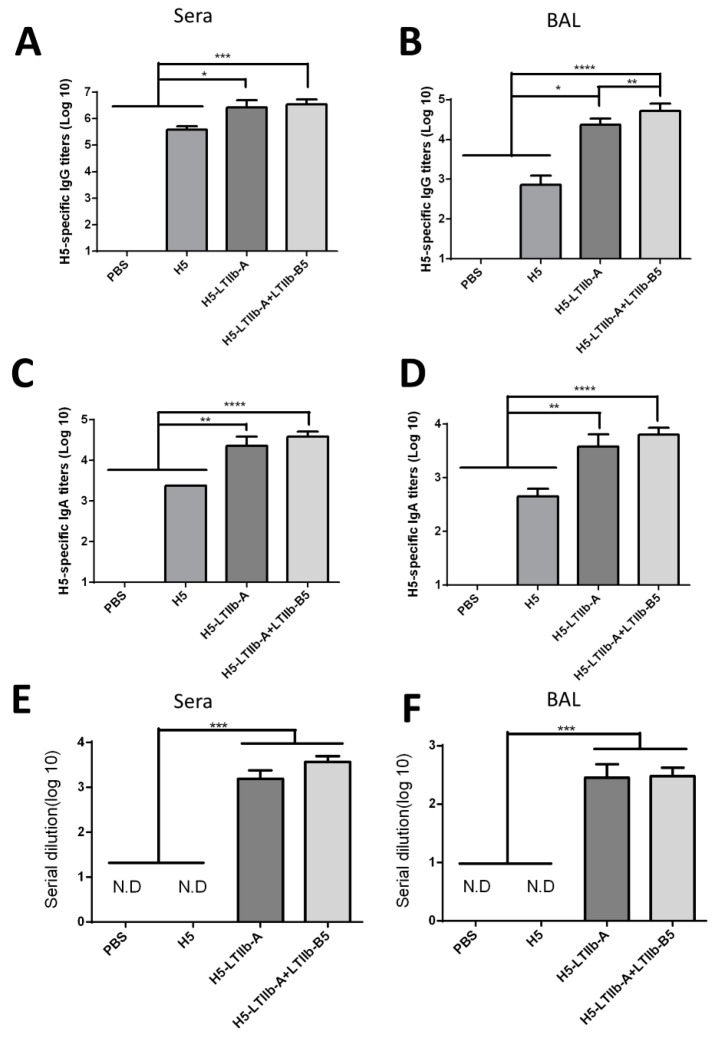
Antibody responses elicited by intranasal immunizations with H5-LTIIb-A fusion protein. Groups of BALB/c mice were immunized with phosphate buffered saline (PBS), H5 protein (H5), H5-LTIIb-A fusion protein (H5-LTIIb-A), or the combination of H5-LTIIb-A fusion protein and another LTIIb-B5 subunit protein (H5-LTIIb-A+LTIIb-B5). The antibody titers were measured for anti-H5 IgG, IgA, and neutralizing antibodies in serum (**A,C,E**) and BALF (**B,D,F**).

To evaluate whether the H5-LTIIb-A fusion protein effectively elicits the systemic and mucosal immune responses in mammals against H5N1 influenza virus, BALB/c mice (five mice per group) were intranasally administered with three doses of the H5-LTIIb-A fusion protein (10 μg), the recombinant H5 protein (10 μg), the H5-LTIIb-A fusion protein (10 μg) plus the recombinant LTIIb-B5 protein (5 μg), or PBS (as sham control) at 3 week intervals. H5-specific IgG and IgA titers in mouse serum and BALFs were measured by ELISA. Results showed that immunization with either H5-LTIIb-A or H5-LTIIb-A + LTIIb-B5 elicited significantly higher H5-specific IgG titers than H5 alone in mouse sera and BALFs (Figure 3A,B). In addition, H5-specific IgA titers were considerably higher in the H5-LTIIb-A and H5-LTIIb-A + LTIIb-B5 groups compared to those in the H5 group (Figure 3C, D). We further examined the neutralizing ability of antibodies from sera and BALFs fluids using the H5N1 pseudovirus. The titers of neutralizing antibodies were defined as the fold of dilution of the serum or BALF that is required to reduce virus infection by 50%. Both immunized H5-LTIIb-A and H5-LTIIb-A + LTIIb-B5 were able to elicit effective neutralizing antibodies in sera and BALFs against the H5N1 pseudovirus (Figure 3E,F). These results revealed that the H5-LTIIb-A fusion protein induced a stronger humoral immune response in mice.

### 3.4. Intransal Immunization with H5-Ltiib-A Augmented Th1/Th17 Responses at Both Systemic and Mucosal Sites

**Figure 4:**
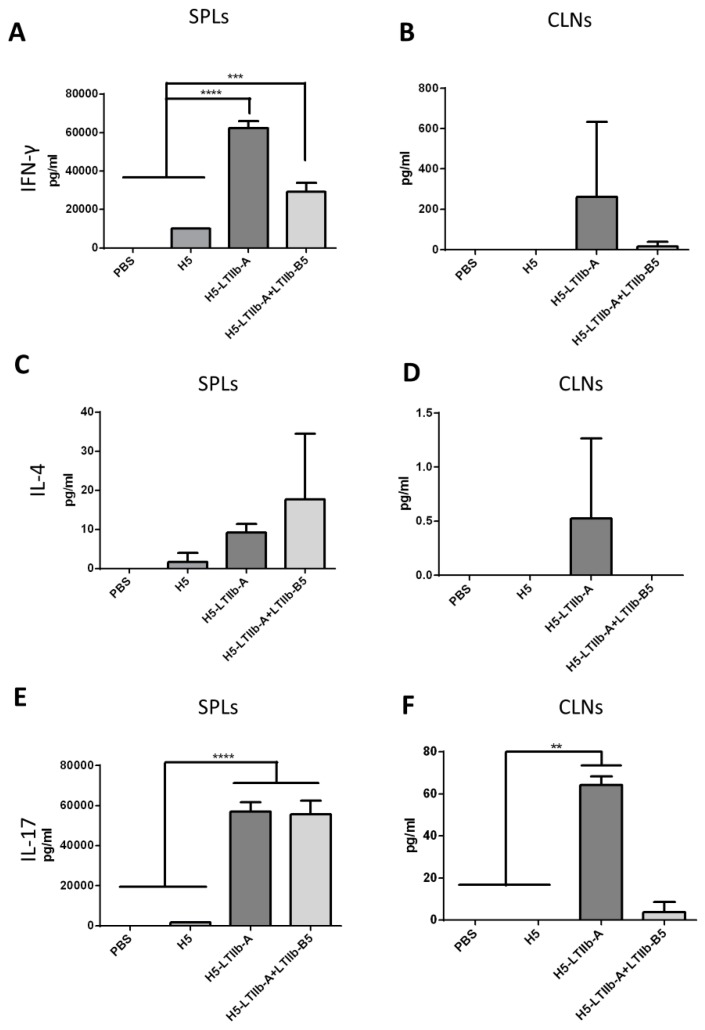
T cell responses elicited by intranasal immunizations with H5-LTIIb-A fusion protein. Groups of BALB/c mice were immunized with phosphate buffered saline (PBS), H5 protein (H5), H5-LTIIb-A fusion protein (H5-LTIIb-A), or the combination of H5-LTIIb-A fusion protein and another LTIIb-B5 subunit protein (H5-LTIIb-A+LTIIb-B5). The levels of IFN-γ-secreted cytokine by the H5-stimulated T cells from spleen (SPLs) and cervical lymph nodes (CLNs) (**A,B**); the levels of IL-4-secreted cytokine by the H5-stimulated T cells from SPLs and CLNs (**C,D**); the levels of IL-17A-secreted cytokine by the H5-stimulated T cells from SPLs and CLNs. Statistical significance was expressed as * *p* < 0.05; ** *p* < 0.01; *** *p* < 0.001; **** *p* < 0.0001.

After the mice were sacrificed, their spleens and cervical lymph nodes were collected and ground, and a fixed number of the cells were cultured in a 24-well plate. A mixture of H5 hemagglutinin peptides was used to stimulate T cells in the spleen and CLNs, and the levels of IFN-γ, IL-4, and IL-17A secreted by Th1, Th2, and Th17 cells, respectively, were measured. IFN-γ was not secreted by the splenocytes of PBS-immunized mice, and a lesser amount of IFN-γ was secreted by the spleen cells of mice immunized with the recombinant H5 proteins. In comparison to these lesser amounts, a threefold increase in IFN-γ secretion was observed in cells from the mice immunized with H5-LTIIb-A + LTIIb-B5, and the secretion of IFN-γ was enhanced by six times in cells from the mice immunized with H5-LTIIb-A (Figure 4A). Similarly, IFN-γ was not secreted by the CLN cells of mice immunized with PBS or H5. A small amount of IFN-γ was secreted by the CLN cells of mice immunized with H5-LTIIb-A + LTIIb-B5, whereas the highest secretion of IFN-γ was observed in cells from the mice immunized with H5-LTIIb-A fusion protein (Figure 4B). IL-4 was not secreted by the splenocytes of PBS-immunized mice, and only trace amounts of IL-4 were secreted by rats immunized with H5. Comparatively, the highest secretion of IL-4 was observed in cells from mice co-immunized with H5-LTIIb-A + LTIIb-B5, and higher IL-4 secretion was also achieved by immunization with H5-LTIIb-A (Figure 4C). No IL-4 secretion was observed in the CLN cells of mice immunized with PBS, H5, or H5-LTIIb-A + LTIIb-B5, whereas about 0.5 pg/mL IL-4 was detected in the immunized H5-LTIIb-A group (Figure 4D). IL-17A was detected in small amounts by the splenocytes of PBS-immunized and H5-immunized mice. In contrast, IL-17A secretion was significantly enhanced in cells from the mice immunized with H5-LTIIb-A alone or H5-LTIIb-A + LTIIb-B5 (Figure 4E). IL-17A was not secreted by the CLNs of mice immunized with PBS or H5. A small amount of IL-17A was secreted by the CLN cells of mice immunized with H5-LTIIb-A + LTIIb-B5, whereas a significant increase in IL-17A secretion was observed in mice immunized with H5-LTIIb-A (Figure 4F). Thus, the H5-LTIIb-A fusion protein significantly induced T cell-related immune responses, particularly IFN-γ and IL-17A cytokine production.

### 3.5. Protection Against H5N1 Influenza Virus Infection in Mice Intransally Immunized with H5-LTIIb-A Fusion Protein

**Figure 5:**
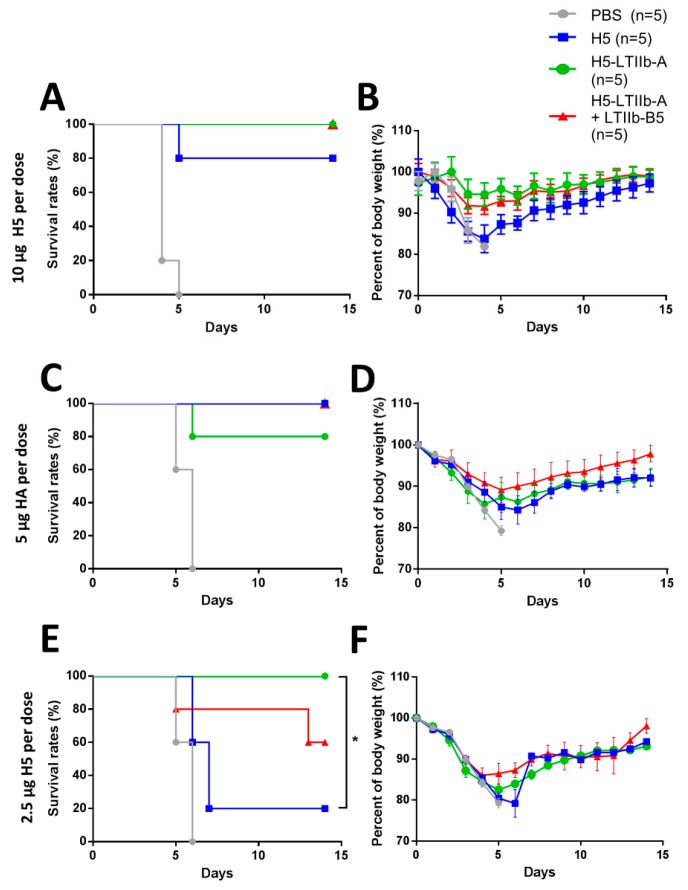
Protection against H5N1 influenza virus infection in mice intranasally immunized with H5-LTIIb-A fusion protein for (**A,C,E**) the survival curves, and for (**B,D,F**) the body weight loss. Groups of BALB/c mice intranasally immunized with PBS, H5 protein (10, 5, or 2.5 μg per dose), H5-LTIIb-A fusion protein (10, 5, or 2.5 μg per dose), or the combination of H5-LTIIb-A fusion protein (10, 5, or 2.5 μg per dose) and 5 μg of recombinant LTIIb-B5 protein for three-dose regimens. Two weeks after the third immunization, each mouse was intranasally injected with a 20-fold lethal dose of H5N1 virus. Statistical significance was expressed as ** *p* < 0.01; *** *p* < 0.001; **** *p* < 0.0001.

The H5-LTIIb-A fusion protein effectively induced H5-specific antibodies and Th1/Th17-related cytokines; thus, we further evaluated whether H5-LTIIb-A is able to elicit predominant protection from H5N1 influenza virus infection. BALB/c mice (five mice per group) were intranasally administered three doses of the H5-LTIIb-A fusion protein (10, 5, or 2.5 μg), the recombinant H5 protein (10, 5, or 2.5 μg), and the H5-LTIIb-A fusion protein (10, 5, or 2.5 μg) in combination with the recombinant LTIIb-B5 protein (5 μg), or PBS at 3 week intervals. Two weeks after the third immunization, each mouse was intranasally injected with a 20-fold lethal dose of H5N1 virus. Using a 10 or 5 μg dosage of the antigen protein, all the PBS-immunized mice died at day 5 or 6 after H5N1 viral challenge, whereas a survival rate greater than 80% at day 14 post-virus infection was observed in mice immunized with H5, H5-LTIIb-A, or H5-LTIIb-A + LTIIb-B5 (Figure 5A,C). No significant difference in the survival rates was found between these three groups. There was only 10% or less reduction of body weight after immunization with either H5-LTIIb-A or H5-LTIIb-A + LTIIb-B5 when using 10 μg antigen (Figure 5B), whereas the mice only immunized by H5-LTIIb-A + LTIIb-B5 retained more than 90% of relative body weight after immunization with 5 μg antigen (Figure 5D).

However, when the dosage of antigen for i.n. administration was reduced to 2.5 μg, there was a significant difference in the survival rates between the mice immunized with H5 or H5-LTIIb-A. The survival rate of mice immunized with H5 was barely 20% at day 7 after virus injection, the survival rate of mice immunized with H5-LTIIb-A was 100% at day 14 after virus injection, and the survival rate of mice co-immunized with the H5-LTIIb-A + LTIIb-B5 was 80% at day 13 after virus infection (Figure 5E). All immunization groups lost less than 90% of relative body weight at day 4 or 5; however, the surviving mice restored their body weight to more than 90% at day 7 after immunization with H5-LTIIb-A + LTIIb-B5, and at day 10 after immunization with H5-LTIIb-A alone (Figure 5F). These results showed that intranasal administration of the H5-LTIIb-A fusion protein resulted in significantly increased survival rates and effective protection against H5N1 influenza virus even after using a lower dosage of antigens. Moreover, the combination of H5-LTIIb-A and LTIIb-B5 exhibited a lower effect on body weight compared with the H5 protein alone.

### 3.6. Intransal Immunization with H5-LTIIb-A Fusion Protein in Chickens

**Figure 6:**
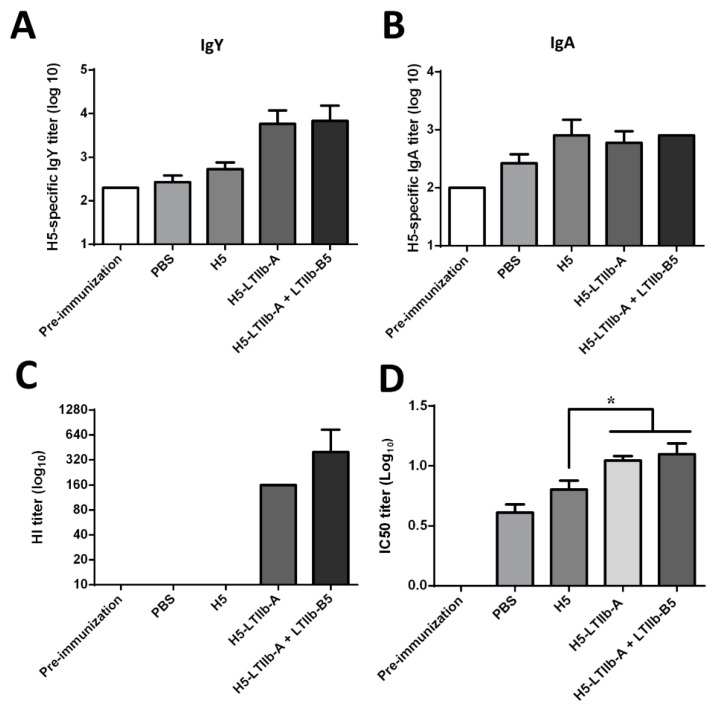
Intransal immunization with H5-LTIIb-A fusion protein in chickens. Chicks (three per group) were intranasally administered with three doses of H5-LTIIb-A fusion protein (10 μg), H5 protein (10 μg), the H5-LTIIb-A fusion protein (10 μg) in combination with the LTIIb-B5 protein (5 μg), or PBS (as sham control). (**A**) H5-specific IgY titer, (**B**) H5-specific IgA titer, (**C**) viral hemagglutinin inhibition (HAI) titer, and (**D**) neutralizing antibody titer of antisera from the immunized chickens. Kaplan–Meier analysis was adopted for survival analysis, with the statistical significance * *p* < 0.05.

To examine whether the H5-LTIIb-A fusion protein effectively elicits a systemic immune response in birds against influenza virus, chickens (three per group) were administered, via intranasal injection, three doses of the H5-LTIIb-A fusion protein (10 μg), the recombinant H5 protein (10 μg), the H5-LTIIb-A fusion protein (10 μg) in combination with the recombinant LTIIb-B5 protein (5 μg), or PBS (as sham control), followed by determination of the H5-specific IgY and IgA levels in chick serum using ELISA. The time interval between each of the immunizations was approximately two weeks. Immunization with H5 slightly increased the serum IgY level as compared to that before immunization; however, immunization with H5-LTIIb-A fusion protein increased the levels of IgY. Similarly, the combination of the H5-LTIIb-A fusion protein and the recombinant LTIIb-B5 protein also induced higher levels of IgY (Figure 6A). Immunization with either the recombinant H5, H5-LTIIb-A, or H5-LTIIb-A + LTIIb-B5 led to a slight increase in the serum IgA level as compared to that before immunization (Figure 6B). The results indicated that i.n. administration of the H5-LTIIb-A fusion protein to birds increased the levels of H5-specific IgY and IgA antibodies. 

### 3.7. Induction of Influenza Virus Neutralizing Antibodies in Chickens by Intransal Immunization with H5-LTIIb-A Fusion Protein

The HI assay was used to detect the presence of antibodies in chick serum that inhibited the hemagglutination of the H5N1 influenza virus. In addition, the titers of serum-neutralizing antibodies against the H5N1 influenza virus were determined using PRNT assay. In the HI assay, chicks did not produce HI antibodies after immunization with H5. However, hemagglutination induced by the H5N1 influenza virus was readily inhibited by at least a 40-fold diluted serum that was obtained after immunization with H5-LTIIb-A or H5-LTIIb-A + LTIIb-B5 (Figure 6C). This result indicated that administration of H5-LTIIb-A fusion protein effectively induced antibodies in chickens that inhibited the hemagglutinin of H5N1 influenza virus. On the basis of the neutralization curves of H5N1 influenza virus-neutralizing antibodies in chicken serum, the antiserum obtained after immunization with H5-LTIIb-A or H5-LTIIb-A + LTIIb-B5 exhibited significantly higher H5N1 virus-neutralizing activity than that obtained after immunization with H5 alone (Figure 6D). The results showed that administration of the H5-LTIIb-A fusion protein elicited a significantly higher level of anti-H5N1 influenza virus-neutralizing antibodies in chickens.

## 4. Discussion

In this study, the intranasal administration of the H5-LTIIb-A fusion protein elicited H5-specific humoral immune responses in mice and chickens, including the production of antigen-specific and influenza virus-neutralizing IgG and IgA in serum and BALFs in mice, as well as the production of neutralizing antibodies against H5N1 virus in chickens. Moreover, H5-LTIIb-A, which stimulated the secretion of Th1/Th17-related cytokines, such as IFN-γ and IL-17A, in splenocytes and CLNs exhibited induction of the cell-mediated immune responses in systemic and mucosal immunity. Above all, the lower dosage of H5-LTIIb-A was sufficient to completely protect mice from H5N1 virus infection. Consequently, the H5-LTIIb-A fusion protein provided effective protection against the highly pathogenic avian influenza H5N1 virus, suggesting LTIIb-A can be utilized as an antigen carrier for development of other mucosal protein-based vaccines.

The roles played by adjuvant are most critical for the development of mucosal subunit vaccines for both mucosal and systemic immune induction to elicit more effective protective immunity [26,27]. Cholera toxins or heat labile enterotoxins have been commonly used as potentially mucosal adjuvants [28,29,30]. To improve the antigen processing and presentation for the antigen, a good strategy is to conjugate (or fuse genetically) antigen proteins with adjuvant molecules as the single associated antigen-adjuvant molecules to the same antigen presenting cells for the elicitation of more effective adaptive immune responses [31]. For instance, *Salmonella typhimurium* flagellin, a TLR-5 agonist, was fused with the M2 ectodomain (M2e) of influenza A virus to increase immunogenicity and elicit strong M2e-specific antibodies in humans [32,33]. Here, HPAI H5 protein fused with LTIIb-A subunit was demonstrated to increase the immunogenic potency and effectiveness of the H5 antigen in mice and chickens via an intranasal delivery route. Our results are in agreement with a previous finding that the A subunit alone was capable of activating dendritic cells and enhanced immune responses to multiple antigens following intranasal immunization [16]. Because the AB5 structure of LTIIb is composed of the A subunit (LTIIb-A) and the pentameric B subunits (LTIIb-B5) [9], the B subunit was considered as a non-toxic and safer mucosal adjuvant [21]. However, we constructed the fusion proteins of the pentameric LTIIb-B5 with the trimeric H5, but the complex fusion proteins could not be obtained successfully by baculovirus-insect cell expression. This was likely due to the assembly for the pentameric LTIIb-B5 structure folded with trimeric HA to be correctly folded due to their structure restrictions.

We observed that only H5-LTIIb-A was able to induce NF-κB-driven luciferase in TLR-2/1-transfected HEK 293A cells (Figure 2C). LTIIb-B5, but not LTIIb holotoxin, is well-known to activate the TLR-2/1 signaling pathway [13,34]. To date, however, no report has demonstrated whether the A subunit of LTIIb alone influences or engages TLR-2/1 binding and downstream signaling. Recombinant LTIIb-A protein from *E. coli* expression system also triggered the increase of NF-κB signaling, as observed by TLR-2/1 functional assay (data not shown). Using an insect–cell expression system, the H5-LTIIb-A fusion protein, rather than the H5 protein, triggered NF-κB activation, which further ruled out the increase of NF-κB signaling due to the contamination of other bacterial substances. H5-LTIIb-A was at least a specific agonist to the TLR-2/1 receptor instead of the TLR-5 receptor. We also observed that combination of H5-LTIIb-A and LTIIb-B5 triggered stronger NF-κB activation than diminishing the LTIIb-B5-induced NF-κB signals. We expected that H5-LTIIb-A may somewhat associate with LTIIb-B5 to mimic the AB_5_ structure, which prevents the upper region of LTIIb-B5 from binding to the TLR-2/1 receptor [13]. In contrast, there was no mutual interference between H5-LTIIb-A and LTIIb-B5 in the TLR-2/1 signaling pathway. H5-LTIIb-A further induced more prominent NF-κB activation at a constant concentration of LTIIb-B5. These results revealed that H5-LTIIb-A is a potential adjuvant and exhibits additional immunogenicity in conjugation with the LTIIb-B5 adjuvant.

Following the intranasal immunization of H5-LTIIb-A with or without LTIIb-B5, both sera and BALFs of mice effectively elicited neutralizing antibodies and induced similar levels of IgG and IgA besides that of IgG in BALFs. In cell-mediated immune responses, however, the additional incorporation of LTIIb-B5 for intranasal H5-LTIIb-A immunization was found to suppress the production of IFN-γ by splenocytes and CLNs, as well as the production of IL-4 and IL-17A by CLNs (Figure 4). It has been shown that there are distinctive immunomodulatory and inflammatory properties for the LTIIa holotoxin and its LTIIa-B subunit following intradermal immunization [35]. Mice immunized with the B subunit pentamer had the reduced activation of antigen-specific T cells, which was mainly restricted to IL-5 secretion by T heper cells, as compared to the LTIIa holotoxin [35]. Moreover, the A subunit of LTIIb can inhibit LTIIb-B5-mediated NF-κB activation and TNF-α induction by cAMP-dependent and cAMP-independent mechanisms [14]. In our study, the presence of LTIIb-A did not interrupt the interaction between LTIIb-B5 and the TLR-2/TLR-1 receptor (Figure 2C). Presumably, neither the blocking of the TLR-2/1 binding site of LTIIb-B5 nor competition for its binding to TLR-2/1 receptor is a possible mechanism to suppress the T cell response. Therefore, more insight investigation is needed to clarify the mechanisms for LTIIb-B5 to suppress T cell response elicited by H5-LTIIb-A mucosal immunization.

The H5-LTIIb-A fusion protein exhibited an additional adjuvant effect by itself, and the presence of LTIIb-B5 became dispensable for increasing the abundance of antigen-specific antibodies and neutralizing antibodies. However, mice immunized with the LTIIb-B5 adjuvant exhibited earlier recovery of body weight after H5N1 viral challenge (Figure 5B,D,F), and reduced production of pro-inflammatory cytokines, such as IL-17A in CLNs (Figure 4F). A study reported that mice with Th17 signaling induced by intranasal immunization exhibited a detrimental response against influenza infection [36]. In addition, mice that were intranasally pre-exposed to a Th17-inducing LTIIb holotoxin exhibited aggravated lung pathology after influenza A/PR/8/34 infection [37]. Hence, our results revealed that the combination of H5-LTIIb-A and LTIIb-B5 adjuvants possibly prevented the side effect of Th17 hypercytokemia to recover mouse weight upon viral infection. Thus, combined vaccination for influenza through the intranasal route is safer and more effective.

## 5. Conclusions

Intranasal administration of the H5-LTIIb-A fusion protein elicited H5-specific humoral immune responses in mice and chickens, and also stimulated the H5-specific Th1 and Th17 cell-mediated immune responses in systemic and mucosal sites. This study employed the novel adjuvants of LTIIb for the development of a new generation of mucosal vaccines against highly pathogenic avian influenza viruses. 

## 6. Patents

Wu, S.C. et al. Title: Influenza mucosal vaccine composition and preparation and application thereof. Taiwan Patent I 646973; U.S. Application No.: 16/053,963.
